# Anti-Inflammatory Effects of Agrimoniin-Enriched Fractions of *Potentilla erecta*

**DOI:** 10.3390/molecules21060792

**Published:** 2016-06-18

**Authors:** Julia Hoffmann, Federica Casetti, Ute Bullerkotte, Birgit Haarhaus, Jan Vagedes, Christoph M. Schempp, Ute Wölfle

**Affiliations:** 1 Department of Dermatology, University Medical Center, 79104 Freiburg, Germany; julia.hoffmann@uniklinik-freiburg.de (J.H.); federica.casetti@@uniklinik-freiburg.de (F.C.); usfreiburg@gmx.de (U.B.); birgit.haarhaus@uniklinik-freiburg.de (B.H.); christoph.schempp@uniklinik-freiburg.de (C.M.S.); 2 ARCIM Institute, 70794 Filderstadt-Bonlanden, Germany; j.vagedes@arcim-institute.de

**Keywords:** *Potentilla erecta*, agrimoniin, COX-2, PGE_2_, UVB, EGFR

## Abstract

*Potentilla erecta* (PE) is a small herbaceous plant with four yellow petals belonging to the Rosaceae family. The rhizome of PE has traditionally been used as an antidiarrheal, hemostatic and antihemorrhoidal remedy. PE contains up to 20% tannins and 5% ellagitannins, mainly agrimoniin. Agrimoniin is a hydrolyzable tannin that is a potent radical scavenger. In this study we tested the anti-inflammatory effect of four PE fractions with increasing amounts of agrimoniin obtained by Sephadex column separation. First, we analyzed in HaCaT keratinocytes the expression of cyclooxygenase-2 (COX-2) induced by ultraviolet-B (UVB) irradiation. As COX-2 catalyzes the metabolism of arachidonic acid to prostanoids such as PGE_2_, we also measured the PGE_2_ concentration in cell culture supernatants. PE inhibited UVB-induced COX-2 expression in HaCaT cells and dose-dependently reduced PGE_2_. The PE fraction with the highest agrimoniin amount (PE4) was the most effective in this experiment, whereas fraction PE1 containing mainly sugars had no effect. PE4 also dose dependently inhibited the phosphorylation of the epidermal growth factor receptor (EGFR) which plays a crucial role in UVB-mediated COX-2 upregulation. A placebo-controlled UV-erythema study with increasing concentrations of PE4 demonstrated a dose dependent inhibition of UVB-induced inflammation *in vivo*. Similarly, PE4 significantly reduced UVB-induced PGE_2_ production in suction blister fluid *in vivo*. In summary, PE fractions with a high agrimoniin content display anti-inflammatory effects *in vitro* and *in vivo* in models of UVB-induced inflammation.

## 1. Introduction

*Potentilla erecta* (PE) is a small plant with yellow petals that belongs to the family of Rosaceae. The rhizome of PE contains up to 20% tannins [1]. Tannins are astringent polyphenols that bind and precipitate proteins. They are widely distributed in the plant kingdom and are located mainly in vacuoles or in cell walls of plants. They do not interfere with plant metabolism but are probably involved in protection against animals, pathogens or freezing. The surface of these molecules is covered with numerous hydroxyl groups which can interact with reactive moieties on proteins or polysaccharides. Thus tannins can tighten damaged membranes and restore the barrier function of mucous membranes. Tannin-rich plant extracts such as oak bark, hamamelis bark, blueberry fruits, green tea and the rhizomes of PE and ratanhia have been traditionally used as antidiarrheal, anti-ulcerogenic [2], hemostatic and antihemorrhoidal remedies and for wound-healing [1]. PE is spread in temperate, arctic and alpine zones of the Northern hemisphere [1]. Because of their aromatic ring structures its tannins can act as UV-protective screen and are very good radical scavengers [1].

Tannins are grouped into hydrolyzable tannins, consisting of gallic acid and/or ellagic acid and sugar moieties, and condensed tannins or proanthocyanidins, composed of flavanols, especially catechin, forming oligo- to polymeric molecules such as epigallocatechin gallate (EGCG) [3]. The rhizome of PE contains a high amount of hydrolyzable tannins, the dominating of which is agrimoniin, a dimeric ellagitannin with a molecular weight of 1862 D. Agrimoniin was shown by Kanoh to be a very good inhibitor of oxidative stress-induced histamine release from rat peritoneal mast cells [4]. Agrimoniin was about 20 times more effective than EGCG, the main tannin of green tea, and comparable to the best anti-allergic drug Azelastine. Lund, Vennat, Bos and colleagues found that agrimoniin (or an agrimoniin-rich acetonic PE fraction) had also outstanding capacities as an antioxidant, astringent, and elastase inhibitor [5,6,7]. From these results it seemed promising to further investigate the potential use of PE extract and agrimoniin in treatment or prevention of inflammatory skin diseases. Because agrimoniin represents less than 2% of the dry weight in PE [8] we enriched agrimoniin in PE extracts to test if agrimoniin indeed increases the anti-inflammatory effect of PE extracts compared to the tannins from the other fractions.

## 2. Results

### 2.1. Fractionation of PE Extract

**Figure 1:**
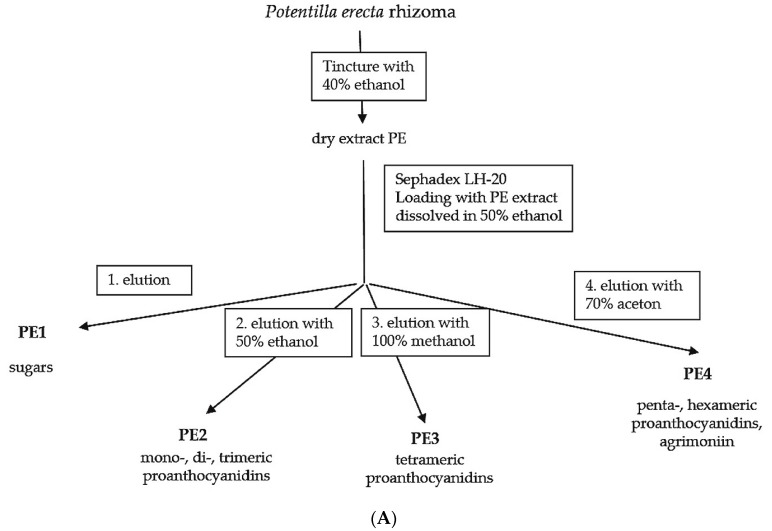

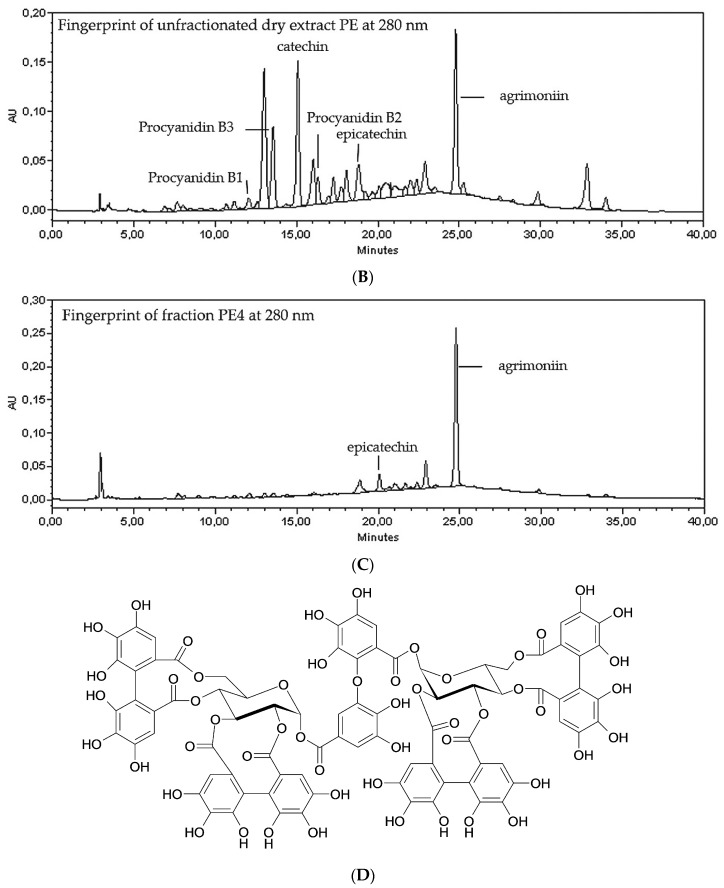
Ethanolic PE dry extract was fractionated by Sephadex LH20 column separation to obtain an ellagitannin-rich fraction (PE4). (**A**) Scheme of the PE fractionation. The mentioned components were identified according to the protocol of Vennat and colleagues [7] and agrimoniin was additionally identified. The graphs show a representative HPLC fingerprint, of the unfractionated PE extract (**B**) and the fraction PE4 (**C**) that contains more than 50% (*w*/*w*) agrimoniin (HPLC channel 280 nm); (**D**) Structural formula of agrimoniin.

Dried crude PE extract were dissolved in 50% ethanol (*v*/*v*) (mobile phase 1) and fractionated via Sephadex LH20^®^ column (HWI, Rülzheim, Germany) with further mobile phases of decreasing polarity: ethanol 50% (*v*/*v*), then methanol 100% and finally acetone 70% (*v*/*v*). The elutions were controlled by thin-layer chromatography according to the method from Vennat and colleagues [7]. These authors identified fractions of different molecular weight and attributed them to proanthocyanidins of different polymerization degrees (Figure 1A–C). Furthermore we detected in PE4 a single prominent ellagitannin of high molecular weight with a concentration of about 50%. This compound was identified as agrimoniin (MW: 1895 D, Figure 1D).

### 2.2. Inhibition of UVB- Induced COX-2 Expression and PGE_2_-Production

**Figure 2:**
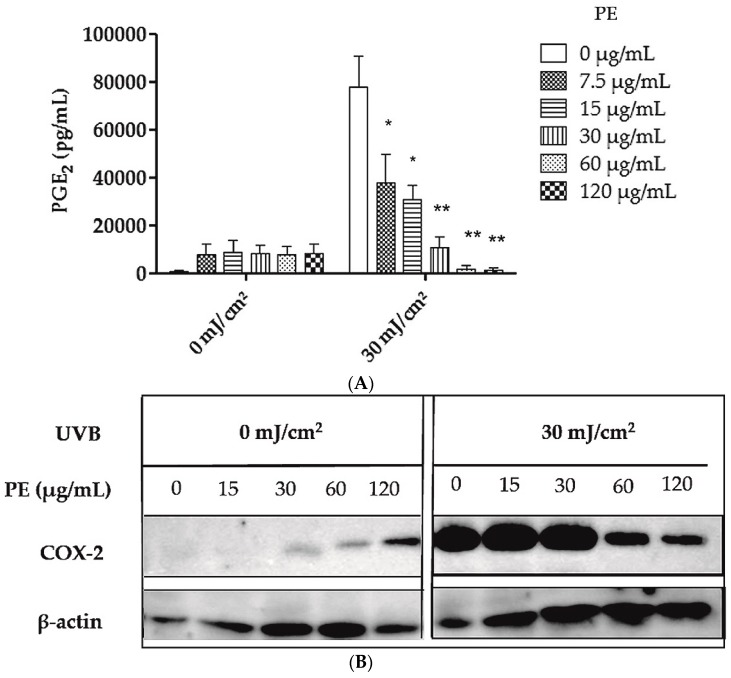
Effect of unfractionated PE extract on UVB-induced COX-2 expression and PGE_2_ production in HaCaT keratinocytes. HaCaT cells were sham irradiated or irradiated with 30 mJ/cm^2^ UVB. (**A**) After irradiation the cells were treated with PE extract for 24 h before the PGE_2_ concentration was measured in the cell culture supernatants. Data are expressed as mean ± SD of three independent experiments and the statistical significance was determined compared to the irradiated, untreated control (* *p* < 0.05; ** *p* < 0.01); (**B**) After irradiation the cells were treated with PE extract for 24 h. Then the cells were lysed and analyzed for COX-2 expression by western blotting. β-actin served as loading control.

HaCaT keratinocytes were sham-irradiated or irradiated with 30 mJ/cm^2^ UVB in the presence or absence of different concentrations of PE extract from 7.5 µg/mL up to 120 µg/mL. PE extract dose-dependently inhibited the UVB-induced upregulation of PGE_2_ in human keratinocytes, while PE extract alone only induced background expression of PGE_2_ (Figure 2A). The half maximal anti-inflammatory concentration EC_50_ was 24.2 µg/mL. Western blotting revealed that PE extract dose-dependently inhibits UVB induced COX-2 upregulation at concentrations of 60 μg/mL and higher while β-actin demonstrated equal protein loading (Figure 2B). COX-2 is induced by UVB radiation and catalyzes the metabolism of arachidonic acid to prostanoids such as PGE_2_ [9].

In subsequent experiments the anti-inflammatory effect of 4 PE fractions with increasing amounts of agrimoniin was investigated with 25 µg/mL (the EC_50_ value of the unfractionated dry extract PE) and a 10 times higher dose (250 µg/mL) to be able to detect also less inhibitory effects of the fractions.

**Figure 3:**
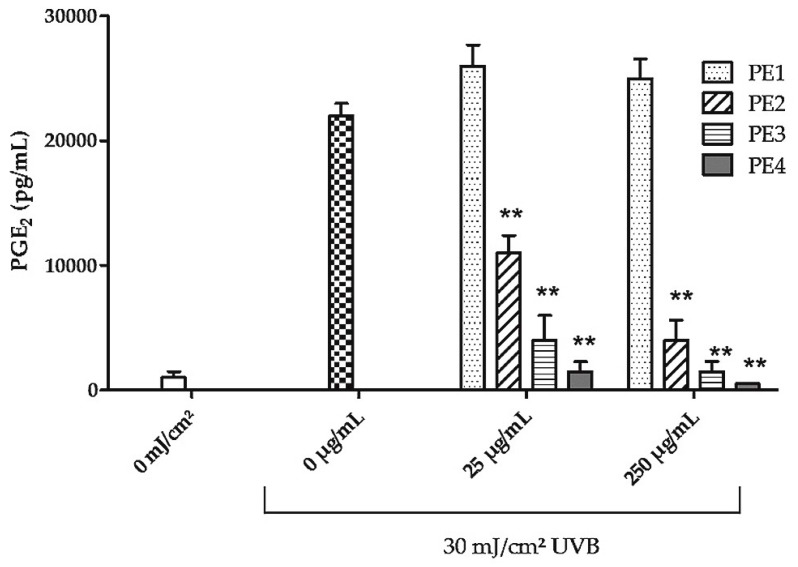
Effect of PE fractions on UVB-induced PGE_2_ production in keratinocytes. After irradiation with 30 mJ/cm^2^ HaCaT cells were treated for 24 h with PE1, PE2, PE3 or PE4 that were obtained by Sephadex LH20 column separation. The concentration of PGE_2_ was measured in the cell culture supernatants. Data are expressed as mean ± SD of three independent experiments and the statistical significance was determined compared to the irradiated, untreated control (** *p* < 0.01).

PE1, that contains mainly sugars, showed no inhibitory effect on UVB-induced PGE_2_ expression. PE2–PE4 showed increasing inhibition of UVB-induced PGE_2_ production. However, PE4 was the most effective fraction and showed already complete inhibition of COX-2 expression at a concentration of 25 µg/mL (Figure 3).

**Figure 4:**
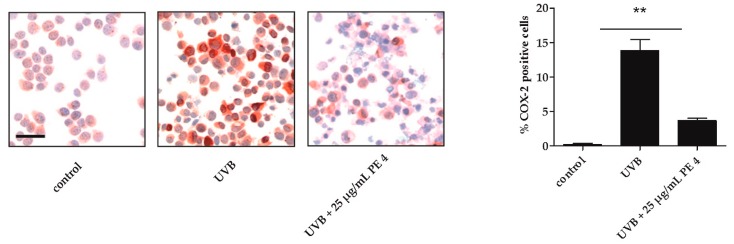
Effect of agrimoniin-enriched fraction PE4 on UVB-induced COX-2 expression. HaCaT cells were irradiated with 30 mJ/cm^2^ UVB and treated with 25 µg/mL PE4. Non-irradiated, untreated cells served as control. Subsequently, the cells were centrifuged on slides and COX-2 expression was measured. The graph shows the percentage of COX-2 positive cells per 100 calculated cells in two staining approaches. The bar corresponds to 50 µm. Data are expressed as mean ± SD and the statistical significance was determined compared to the irradiated, untreated control (** *p* < 0.01).

Then we tested if PE4 also inhibits UVB-induced COX-2 expression. To address this issue, HaCaT keratinocytes were irradiated with UVB and stained for COX-2 expression using an anti-COX-2 antibody. The percentage of COX-2-positive cells was significantly reduced (Figure 4).

**Figure 5:**
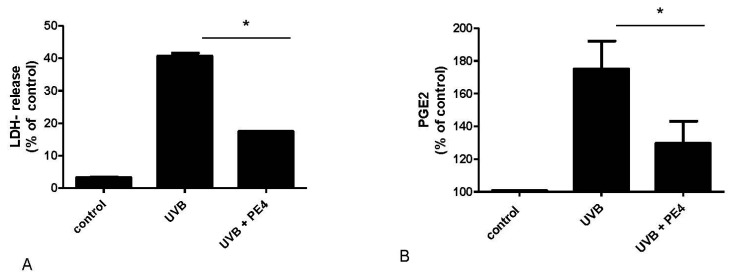
Effect of agrimoniin-enriched fraction PE4 on UVB-induced cell death *in vitro* and on UVB-induced PGE_2_ release *in vivo*. (**A**) HaCaT cells were incubated for 15 min with PE4 (25 µg/mL) before irradiation with 30 mJ/cm^2^ UVB. After 12 h LDH-release was measured to test cytotoxicity of PE4 in HaCaT cells after irradiation. Non-irradiated and untreated cells served as control. Triton X-100 served as positive control and corresponds to 100% LDH release; (**B**) Test areas on the volar skin of healthy volunteers were irradiated with 1.5 MED UVB before treatment for 30 min with 5% PE4. Non-irradiated and untreated test area served as control. After 18 h suction blisters were raised and the blister fluid was analyzed for PGE_2_ concentration. Data are expressed as mean ± SD of three independent experiments (* *p* < 0.05).

To analyze if fraction PE4 can also protect keratinocytes from UVB-induced cell death, HaCaT cells were incubated for 24 h with PE4 (25 µg/mL) before UVB irradiation. Subsequently, the amount of lactate dehydrogenase (LDH) was measured in the supernatants. LDH is a stable cytoplasmic enzyme which is present in all cells but only released when the plasma membrane is damaged. PE4 significantly reduced UVB-induced LDH release (Figure 5A).

UVB-induced PGE_2_ synthesis was also assessed in suction blisters of human volunteers *in vivo*. Compared to the untreated control, PE4 significantly decreased PGE_2_ concentrations in the blister fluid of the volunteers (Figure 5B).

### 2.3. Inhibition of EGF-Induced EGFR Phosphorylation

**Figure 6:**
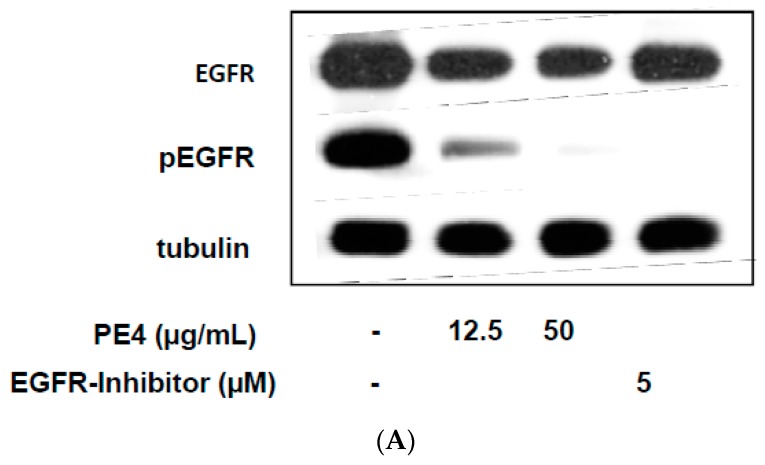

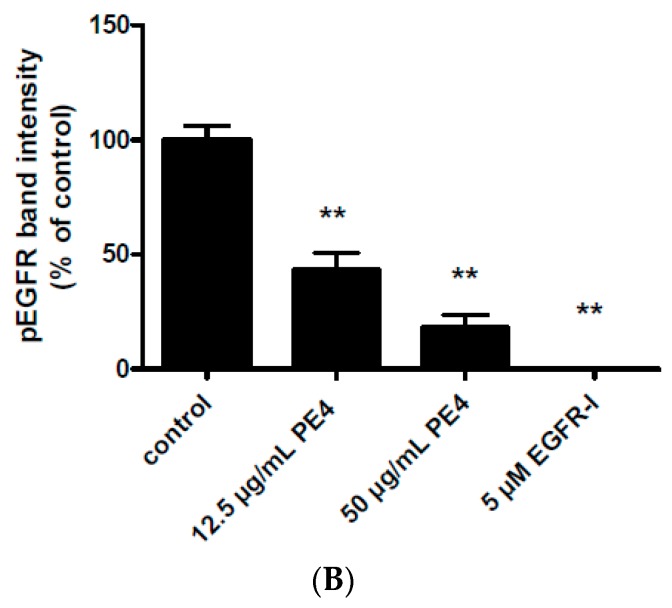
Inhibition of EGFR phosphorylation in A431 cells. A431 cells were treated for 15 min with PE4 at a concentration of 12.5 and 50 µg/mL or with the *EGFR* tyrosine kinase *inhibitor (EGFR-I)* AG1478 (5 µM). Untreated cells served as control. Subsequently the cells were stimulated for 5 min with 50 ng/mL EGF. (**A**) Afterwards the cells were lysed and prepared for western blotting with anti-EGFR, anti-phosphoEGFR (pEGFR) and anti-β actin antibodies; (**B**) The graph shows the band intensity of the western blot. The pEGFR/tubulin quotient was equaled to 100%. Data are expressed as means ± SD of three independent experiments and the statistical significance was determined compared to the untreated control (** *p* < 0.01).

UVB-induced COX-2 expression is mediated by epidermal growth factor receptor (EGFR) phosphorylation and activation of the MAP-kinase pathway [9]. UVB was reported to lead to EGFR phosphorylation by inhibiting protein tyrosine phosphatases. This effect is low and short-lived compared with EGF-induced phosphorylation [10]. Therefore we used the EGF stimulated human squamous carcinoma cell line A431 with a high expression of EGFR. Western blotting of protein extracts showed that PE4 dose dependently inhibits EGFR phosphorylation with the two concentrations 12.5 and 50 µg/mL. 50 µg/mL PE4 was almost as effective as the *EGFR* tyrosine kinase *inhibitor AG1478* that completely blocked EGFR phosphorylation (Figure 6A,B).

### 2.4. Inhibition of UVB-Induced Erythema in Vivo

**Figure 7:**
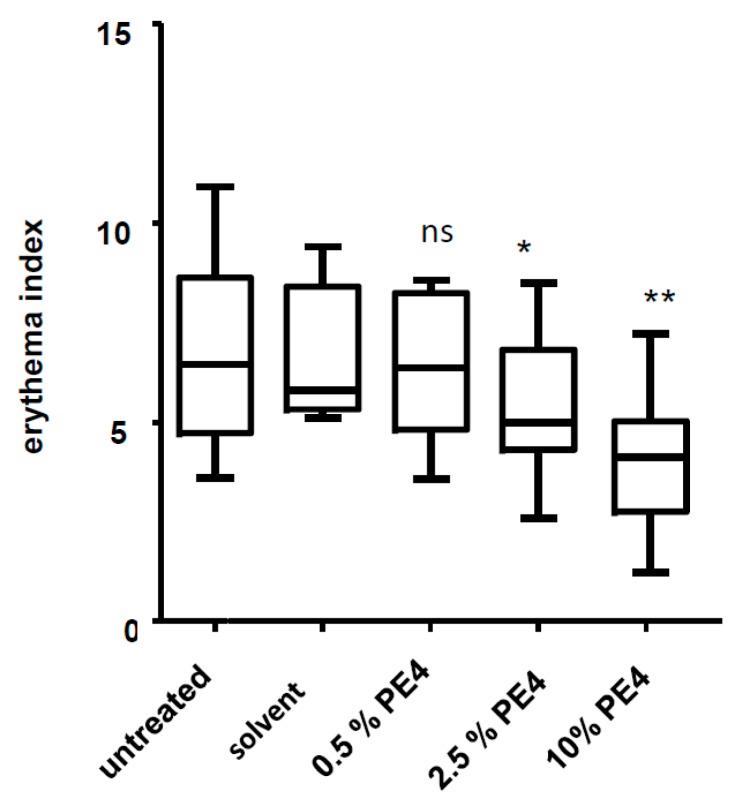
Inhibition of UVB-induced erythema in human skin *in vivo*. Test areas on the back of 8 volunteers were irradiated with 1.5 minimal erythema doses (MED). Subsequently the test areas were occlusively treated for 48 h with the vehicle or the indicated concentrations of PE4. Data are expressed as mean ± SD and the statistical significance was determined compared to the solvent control (* *p* < 0.05; ** *p* < 0.01).

The anti-inflammatory effect of PE4 was confirmed in a placebo-controlled UV-erythema study in healthy volunteers (Figure 7).

The used concentrations of PE4 were 0.5% (5 mg/mL), 2.5% (25 mg/mL) and 10% (100 mg/mL). These concentrations are 1000 times higher compared to those used *in vitro*, because in our experience 500–1000 times higher concentrations have to be used *in vivo* compared to *in vitro* experiments due to the fact that the skin is a penetration barrier.

## 3. Discussion

Tannins from green tea, oak bark, hamamelis or PE have been traditionally used for the treatment of inflammatory skin diseases and wounds. Hsu and colleagues described that tannins may interfere with inflammatory processes in the skin [11]. In the present study we tested the anti-inflammatory effect of crude PE extract as well as 4 PE fractions with increasing agrimoniin concentration. Models of UVB induced inflammation *in vitro* revealed that already the crude PE extract has an anti-inflammatory effect as shown by inhibition of UVB- induced COX-2 expression and PGE_2_-production. This fits to findings of Tunon and colleagues who studied 52 plants based on literature search and found that PE had a strong COX-2 inhibitory property in a cell free system [12]. Other *Potentilla* species such as *P. recta* (not to be confused with *P. erecta*) and *P. reptans* have also been investigated for their UV protective, anti-microbial, radical scavenging, anti-inflammatory and anti-tumor effects [1,13,14,15,16]. In our setting agrimoniin turned out to be the major anti-inflammatory compound of PE. In addition to our findings Bazylko and colleagues described a strong ROS scavenging effect of agrimoniin and *Potentilla recta* extract [17]. The anti-oxidant effect of ellagitannins is directly associated with the degree of hydroxylation [18] and explains why agrimoniin as a large molecule (1862 Da) with numerous hydroxyl groups has a strong anti-oxidant effect. Mota and colleagues assumed already 30 years ago that the interaction of tannins with cell membranes contributes to the anti-inflammatory activity that is related to the astringency of tannins [19]. As agrimoniin has a very potent astringent capacity [20] this may also be a reason for the prominent anti-inflammatory effect of PE4 in our experiments. Besides Pilipovic and colleagues demonstrated an anti-inflammatory effect of *Potentilla malýana* acetone and ethanol extracts in a mouse ear edema model [21]. Furthermore purified agrimoniin was described to be an exceptional good inhibitor of oxidative stress-induced histamine release [4].

Recently, Hrenn and colleagues investigated the effects of nine natural phenolic compounds of different molecule sizes, including agrimoniin, pedunculagin, and EGCG, on human neutrophil elastase (HNE) activity and release [22]. In inflamed or irradiated tissue high HNE activity causes an abnormal degradation of connective tissue proteins, *i.e.*, collagens [23,24]. It has been shown that sun exposure activates HNE and makes collagen fibrils more susceptible to matrix metalloproteinase I (MMP-1) cleavage [25]. Hrenn and colleagues described that agrimoniin is the most potent inhibitor of HNE activity, followed by pedunculagin, another ellagitannin that is also present in PE extracts. In contrast, EGCG was about 30 times less effective than agrimoniin. However, docking experiments revealed that agrimoniin and pedunculagin are too large to fit into the elastase binding site and cannot form specific interactions with binding site residues. Therefore, elastase inhibition by ellagitannins may be due to coverage of the hydrophobic S1 pocket and active site, or may occur in an unspecific manner, as supposed from the existence of the numerous phenolic hydroxyl groups [22]. All these data support our findings that agrimoniin seems to be the most important component that mediates the anti-inflammatory effect of PE.

Furthermore, we could demonstrate that agrimoniin-enriched PE4 inhibits EGFR phosphorylation. As EGFR activation in association with increased COX-2 expression is involved in photocarcinogenesis of keratinocytes [9,26] and EGFR seems to be a key mediator of UVB-induced skin cancer, PE4 might also be effective in skin cancer prevention. This hypothesis is strengthened by the fact that increasing amounts of COX-2 are commonly found in both premalignant and malignant tissues, and overexpression of COX-2 leads to increased amounts of prostanoids in tumors, e.g., PGE2 [27,28,29]. Furthermore PGE_2_ can stimulate cell proliferation and motility while inhibiting immune surveillance and apoptosis [29]. Topical treatment with an agrimoniin-rich PE fraction might therefore be useful to treat early skin cancers and mild actinic keratosis. This is especially interesting as PE extract was not toxic when administered to mice in high dosages [5], and *Potentilla* species are considered to be one of the safest astringents in the treatment of diarrhoea and sore throat [12].

Taken together, our results demonstrate anti-inflammatory effects of agrimoniin-enriched PE extracts that might be useful in the treatment of UV-induced skin damage.

## 4. Experimental Section

### 4.1. Extracts and Chemicals

Pharmacological and clinical experiments were performed with a commercially available PE extract (Tinctura Tormentillae from Caesar & Loretz GmbH, Hilden, Germany) and PE fractions that were obtained by Sephadex column separation using different solvents as described [7,30].

In brief, dried PE extract was obtained by vacuum evaporation of ethanolic PE extract (<50 °C), 100 mL yielding 1 g dry extract (100:1); with respect to dry source material, the drug/extract ratio is 20:1. The fractionation of the PE dry extract was performed by HWI analytics (Rülzheim, Germany). For fractionation of the PE-crude extract 30 g dry extract were dissolved in 200 mL ethanol 50% (mobile phase 1) and fractionated via Sephadex LH20^®^ (HWI) column with mobile phases of decreasing polarity: ethanol 50%, methanol 100%, acetone 70%. Fractions were controlled by thin-layer chromatography (silica gel 60, mobile phase ETAC:HCOOH:CH_3_COOH:H_2_O; 100:11:11:27, UV-detection, vanillin-H_3_PO_4_; 120 °C). This method was adapted without alterations from Vennat and colleagues [7]. They identified fractions of different molecular weight and attributed them to proanthocyanidins of different polymerization degrees. However, our own investigations using HPLC -MS showed that in fraction PE4 a single ellagitannin of high molecular weight dominated with about 50%, and this compound was identified as agrimoniin (MW: 1895 D). Minor compounds were pedunculagin and laevigatin (see Figure 1 in the result section).

The pure substances laevigatin F, pedunculagin and agrimoniin as HPLC marker were kindly provided by Prof. Merfort (Institute of Pharmaceutic Sciences of the Albert-Ludwigs-University of Freiburg, Germany). The following antibodies and concentrations or dilutions were used for immunohistochemical stainings: the monoclonal mouse anti-human COX-2-antibody (1:200 (cytochemical staining); 1:1000 (western blotting)); Santa Cruz Biotechnology, Heidelberg, Germany), the polyclonal goat anti-human β-actin antibody (1:1000, Santa Cruz Biotechnology), the monoclonal mouse anti-human β-tubulin antibody (1:1500, Sigma Aldrich, Taufkirchen, Germany), the polyclonal rabbit anti-pEGFR antibody (1:1000, Merck Millipore, Darmstadt, Germany) and the monoclonal mouse anti-EGFR antibody (1:500, Dako, Hamburg, Germany). The secondary antibodies were the polyclonal goat anti-mouse horseradish peroxidase (HRP) conjugated antibody (1:5000, Biorad, München, Germany) and the polyclonal goat anti-rabbit HRP conjugated antibody (1:5000, Santa Cruz Biotechnology). The secondary antibody multi-link-biotin, the streptavidin- HRP-label were from BioGenex (Hamburg, Germany) and the AEC-substrate were from Thermo Fisher Scientific GmbH, Darmstadt, Germany) and were used according to the manufacturer’s instruction. The primary arachidonic acid (AA), sodium chloride was purchased from Sigma, Phenylmethylsulphonyl fluoride (PMSF) and sodium orthovanadate were from Roche (Mannheim, Germany). For protein determination the bicinchoninic acid protein assay (BCA) (Pierce, Rockford, IL, USA) was used. The EGFR tyrosine kinase inhibitor AG1478 was from abcam (Cambridge, UK).

### 4.2. Cell Culture

The human keratinocyte cell line HaCaT and the human squamous carcinoma cell line A431 were cultured in DMEM (Thermo Fisher Scientific GmbH) containing 10% FCS (Serumed, Merck, Darmstadt, Germany) at 37 °C in a humidified atmosphere with 5% CO_2_.

For PGE_2_ ELISA the cells were seeded in 5 cm Petri dishes and allowed to attach for 24 h. Subsequently, the cell culture medium was replaced by PBS. The cells were then irradiated with UVB. The UVB radiation between 270 and 400 nm, peaking at 310 nm was delivered from 10 fluorescent UVB lamps, Philips TL20W/12 (Philips GmbH, Hamburg, Germany), housed in a UV 800 unit (Waldmann GmbH, VS-Schwenningen, Germany). Cells were irradiated on ice with increasing doses of UVB as indicated. Directly after irradiation PBS was replaced by fresh medium and PE extract was added at the indicated concentrations. Sham irradiated cells were used as untreated control. COX-2 activity was saturated with 30 µM arachidonic acid for 15 min. After 24 h the cell culture supernatants were collected for PGE_2_ determination. Then the cells were harvested and lysed for 10 min on ice using lysis buffer (50 mM TRIS–HCl, 150 mM NaCl, 0.5% NP-40, 5 mM EDTA, 2 mM PMSF, 20 µM sodium orthovanadate) followed by centrifugation for 15 min at 4.000 rpm and 4 °C. Total protein (30 µg of each sample) was subjected to sodium dodecyl sulfat polyacrylamide gel electrophoresis (SDS-PAGE).

### 4.3. PGE_2_-ELISA

PGE_2_ concentrations in the supernatants were analyzed by a high sensitivity PGE_2_ ELISA (R&D systems, Wiesbaden, Germany) according to the manufacturer’s protocol. The results of the ELISAs were measured with an ELISA reader (Sirius HT, Eurofins, Ebersberg, Germany). Data were expressed as mean ± SD of three independent experiments.

### 4.4. Suction Blister Experiments

Test areas on the volar aspects of the forearms of three volunteers were investigated. The study protocol was approved by the ethics committee of the University of Freiburg and written informed consent was obtained from all subjects. Two areas were irradiated with UVB at a dose equivalent to 1.5-fold of the minimal erythema dose (MED), and the third area was not irradiated and served as control. One of the designated areas was treated topically with 5% fraction PE4 in a 66% glycerin/ethanol mixture after irradiation. The other area was left untreated. Eighteen hours after irradiation suction blisters were raised on all areas as previously described [31]. Briefly, sterile suction cups of acrylic plastic were applied with a vacuum of 300–500 mbar, resulting in the formation of four suction blisters. The blister fluid was collected, snap frozen, and stored at −80 °C until used in the PGE_2_ ELISA.

### 4.5. Western Blot

After separation the proteins were transferred electrophoretically to a polyvinylidene fluoride (PVDF) membrane (Millipore GmbH, Schwallbach, Germany) and western blotting was performed as described with the primary monoclonal COX-2 and β-actin antibodies. The antigen-antibody-complexes were detected by the ECL-Plus western blot detection system (Amersham Biosciences, Uppsala, Schweden) using an X-ray film (Hyperfilm, Amersham Pharmacia Biotech, Buckinghamshire, UK). The assay was repeated three times with similar results.

### 4.6. Immunocytochemistry

HaCaT cells were treated for 24 h with 25 µg/mL PE4 after irradiation with UVB. Then the cells were centrifuged on slides and stained with the monoclonal antibody against COX-2. Application of the primary antibody (4 °C, overnight) was followed by incubation with biotinylated swine anti-goat, anti-mouse and anti- rabbit antibody immunoglobulins (1 h, RT), streptavidin conjugated to horseradish peroxidase (20 min, RT), AEC solution as chromogen and hematoxylin counterstaining. H&E staining was performed with hematoxylin and eosin solution from Sigma. Images were taken with a microscope (Carl Zeiss AG, Oberkochen, Germany) equipped with Axiovision software.

### 4.7. Cell Death Assay

Cell death was determined by measuring lactate dehydrogenase (LDH), a stable cytoplasmic enzyme which is present in all cells but only released when the plasma membrane is damaged. The LDH cytotoxicity detection kit from Roche provides a simple and precise colorimetric assay for LDH activity in which in a two-step enzymatic reaction a formazan dye was created that could easily be measured by A_492_. All data are expressed as mean ± SD of at least three independent experiments.

### 4.8. UV Erythema Test

The anti-inflammatory effect of PE4 was determined in 8 healthy volunteers in the UVB erythema test. The study protocol was approved by the ethics committee of the University of Freiburg and written informed consent was obtained from all subjects. Inclusion criteria were healthy, non- smoking persons of both sexes, age >18 years, skin types II and III. Exclusion criteria were skin types I or IV, allergies, skin diseases, photosensitivity, sunbed tanning, metabolic diseases, infections, pregnancy, breast feeding, and participation in other studies during the last 2 months.

After determination of the minimal erythema dose (MED) the irradiation dose was calculated for each volunteer (1.5 MED). Background erythema (T0) was measured in all test areas before treatment using a Mexameter MX 16 (Courage & Khazaka Electronics GmbH, Köln, Germany). The test areas were then irradiated with 1.5 MED. Subsequently, 50 µL of the test preparations were applied on the test areas using Finn chambers (1.8 cm^2^, Hermal, Hamburg, Germany) and were fixed with Fixomull (6 × 4 cm, BSN Medical GmbH, Hamburg, Germany). The PE extract was incorporated at the indicated concentrations into the vehicle (66% glycerol in ethanol). After 48 h the test substances were removed, and after a resting phase of 30 min the test areas were measured a second time (T1) and the erythema index T1-T0 was calculated.

### 4.9. Statistical Analysis

All data were expressed as mean ± SD of three independent experiments. Statistical evaluation was performed using Student’s *t*-test for unpaired observations. Data of the UV erythema test were analyzed using the Wilcoxon test for pairwise comparisons. *p*-values ≤ 0.05 were considered significant and were indicated in the figure by one asterisk (*); *p* < 0.01 was indicated with two asterisks (**).
